# DOES INTERNET USE PROMOTE OLDER ADULTS’ SELF-PERCEPTION OF AGING: SEPARATING WITHIN- AND BETWEEN-PERSON EFFECTS

**DOI:** 10.1093/geroni/igad104.2700

**Published:** 2023-12-21

**Authors:** Kun Wang, Danan Gu

**Affiliations:** Binghamton University, State University of New York, Binghamton, New York, United States; The United Nations, New York City, New York, United States

## Abstract

Previous longitudinal studies indicate a positive causal association between older adults’ Internet use and self-perception of aging (SPA). However, it is unclear whether this positive effect is truly due to the lagged within-person changes in Internet use or due to some stable, trait-like between-person differences. Thus, with between-person differences controlled, this study aimed to 1) examine reciprocal associations of within-person SPA and Internet use and 2) investigate the potential age, gender, race/ethnicity, and education group differences. Data used in this study were from the Health and Retirement Study (T1: 2008; T2: 2012; T3: 2016). Older adults aged 65 and older with normal cognition were included in the analysis (n = 6,666). Random-intercept cross-lagged panel models (RI-CLPM) were applied to separate the within- and between-person effects regarding the associations between SPA and Internet use. Multiple-group analyses were used for testing group differences. Results showed positive carry-over stability of both Internet use and SPA across time. At the within-person level, only one cross-lagged path, Internet use (T1) ♢ SPA (T2), was significant (b = .08, p < .05), indicating that the within-person Internet use change was predictive of within-person SPA change at T2. At the between-person level, SPA and ICT use were positively correlated (r = .16, p < .001). No group differences were identified. This study indicates that the positive effects of Internet use on SPA may be largely due to between-person differences. Internet use may promote SPA to some extent, but effects may plateau after a certain period of time.

